# *In vitro* evaluation of osteoprotegerin in chitosan for potential bone defect applications

**DOI:** 10.7717/peerj.2229

**Published:** 2016-08-23

**Authors:** Soher Nagi Jayash, Najihah M. Hashim, Misni Misran, NA Baharuddin

**Affiliations:** 1 Department of Restorative Dentistry, Faculty of Dentistry, University of Malaya,Kuala Lumpur,Malaysia; 2 Department of Pharmacy, Faculty of Medicine, University of Malaya,Kuala Lumpur,Malaysia; 3 Centre For Natural Products And Drug Discovery (CENAR), Department of Chemistry, Faculty of Science, University of Malaya,Kuala Lumpur,Malaysia; 4 Department of Chemistry, Faculty of Science, University of Malaya,Kuala Lumpur,Malaysia

**Keywords:** Chitosan, Osteoproteogerin, Bone, Normal human periodontal ligament fibroblast, Normal human osteoblast

## Abstract

**Background:**

The receptor activator of nuclear factor kappa-B (RANK)/RANK ligand/osteoprotegerin (OPG) system plays a critical role in bone remodelling by regulating osteoclast formation and activity. OPG has been used systemically in the treatment of bone diseases. In searching for more effective and safer treatment for bone diseases, we investigated newly formulated OPG-chitosan complexes, which is prepared as a local application for its osteogenic potential to remediate bone defects.

**Methods:**

We examined high, medium and low molecular weights of chitosan combined with OPG. The cytotoxicity of OPG in chitosan and its proliferation *in vitro* was evaluated using normal, human periodontal ligament (NHPL) fibroblasts in 2D and 3D cell culture. The cytotoxicity of these combinations was compared by measuring cell survival with a tetrazolium salt reduction (MTT) assay and AlamarBlue assay. The cellular morphological changes were observed under an inverted microscope. A propidium iodide and acridine orange double-staining assay was used to evaluate the morphology and quantify the viable and nonviable cells. The expression level of osteopontin and osteocalcin protein in treated normal human osteoblast cells was evaluated by using Western blot.

**Results:**

The results demonstrated that OPG in combination with chitosan was non-toxic, and OPG combined with low molecular weight chitosan has the most significant effect on NHPL fibroblasts and stimulates proliferation of cells over the period of treatment.

## Introduction

Osteoprotegerin (OPG) is a secretory glycoprotein of the tumour necrosis factor (TNF) receptor, which is highly expressed in adult bone, lung, heart, kidney and placenta (Nagasawa et al., 2002; Baharuddin et al., 2015). The role of OPG in the pathological aspects of bone diseases, such as osteoporosis associated with estrogen deficiency and periodontal disease, has been well established (Bostanci et al., 2007; Koide et al., 2013; Hofbauer et al., 1999).

In the medical field, OPG therapy has been used to reduce bone resorption and to enhance osseous healing (Kostenuik et al., 2001; Bekker et al., 2001); the therapeutic strategies are based on OPG’s potent inhibitory action on osteoclast differentiation and function. In tumour-bearing mice, OPG treatment reduced osteoclast activity.

Bekker et al. (2001) extended gene therapy investigations into human clinical trials by investigating the safety and tolerability of OPG as well as the bone anti-resorptive effects. This study showed that a single dose of OPG rapidly decreased bone resorption in post-menopausal women. Thus, blocking RANKL using OPG may be effective in the treatment of bone diseases characterized by increased bone resorption, such as osteoporosis.

Chitosan is a natural, cationic carbohydrate polymer derived from chitin by partial deacetylation (Ilium, 1998). It has been shown to be biocompatible and biodegradable both for *in vitro* and *in vivo* conditions (Joshi et al., 2012; Coimbra et al., 2011). Chitosan also has high affinity to proteins, adheres well to mucosa and demonstrates antifungal effects; thus, it makes an ideal material for biomedical applications. To our knowledge, there is no study investigating the use of a drug delivery system with a polymer/polysaccharide matrix, such as chitosan, to deliver OPG locally. Therefore, in this study, we attempt to evaluate the cytotoxicity of low, medium and high molecular weights of chitosan (LMW, MMW and HMW respectively) and new combinations of OPG and chitosan (OPG-chitosan complexes) and their *in vitro* effect on normal, human periodontal ligament (NHPL) fibroblast cells and study the effect on bone marker production from normal human osteoblast cells.

## Materials and Methods

### Materials

Low molecular weight (LMW), medium molecular weight (MMW) and high molecular weight (HMW) chitosan and human OPG protein (Recombinant Human OPG; PeproTech, Rocky Hill, New Jersey, USA) were used in this study. Tris buffer (5 mmol L^−1^, pH 7.5) and dimethyl sulfoxide (DMSO) (Fisher Scientific, Leics, UK) were used throughout the experiment. Normal, human periodontal ligament (NHPL) fibroblasts and normal, human osteoblasts were obtained from Lonza (Lonza Inc., Walkersville, MD, USA). Penicillin-streptomycin (Bioscience Ltd, Buckingham, UK), 3-(4, 5-dimethylthiazol-2-yl)-2, 5-diphenyltetrazolium bromide (MTT) propidium iodide and acridin orange (Sigma-Aldrich, St. Louis, MO, USA) were also used.

### Cell culture

NHPL fibroblast cells were cultured and maintained in Dulbecco’s Modified Eagle’s Medium (DMEM; Sigma-Aldrich, St. Louis, MO, USA). The medium was supplemented with 10% foetal bovine serum (FBS) with 1% antibiotics (penicillin-streptomycin) and incubated in 5% CO_2_ at 37 °C. The medium was changed twice a week until a confluent cell monolayer was formed and observed under an inverted microscope.

### Cell viability assay

We evaluated the effect of different molecular weights of chitosan (LMW, MMW and HMW) and OPG on cell viability after treatment. The cultured cells were trypsinized, seeded in 96-well micro plate (8 × 10^3^ cells/well) and incubated at 37 °C in 5% CO_2_ for 24, 48 and 72 h to allow cell attachment as described earlier (Souza et al., 2010). The medium was freshened and treated with serial dilutions of chitosan (100, 50, 25, 12.5, 6, 3, 1.5, μg mL^−1^) and OPG (30, 15, 7.5, 3, 1.5, 0.75, 0.35, 0.19, 0.09, 0.045, 0.024 μg mL^−1^) then incubated for 24, 48 and 72 h. Following incubation, 20 μL of tetrazolium bromide (MTT) (5 mg mL^−1^) solution was added to each well followed by incubation for 4 h. All remaining supernatant was removed and 100 μL of DMSO was added to dissolve the crystal formation. The optical density was measured at a wavelength of 570 nm using a microplate reader (Tecan Infinite M 200 PRO; Tecan, Männedorf, Switzerland).

### Cell proliferation assay

A cell proliferation assay was carried out by using MTT on various concentrations of OPG-chitosan combinations. The different MWs of chitosan (at fixed concentration) combined with high, moderate and low concentrations of OPG were selected from the results of the cell viability assay. NHPL cells (8 × 10^3^/well) were treated with the OPG-chitosan combinations together with control samples (cells without treatment) and incubated for 24, 48 and 72 h. Each sample was assayed in triplicate.

At the end of each incubation period, the previously explained procedure was used in another viability assay to measure the optical density by MTT assay.

### Morphological observation

NHPL cells were seeded into 24-well microtiter plate (with a density 3 × 10^4^ cells/well) and were incubated for 24 h at 37 °C and 5% CO_2_. Then the cells were treated with different MWs of chitosan, combined with 0.024 μg mL^−1^ OPG and incubated for 24, 48 and 72 h. The cellular morphological changes of the treated and untreated cells were observed under a Leica DM IRB (Leica Microsystems, Wetzlar, Germany) inverted microscope and compared with untreated, viable cells.

### Acridine orange and propidium iodide (AOPI) double-staining assay

Acridine orange (AO) and propidium iodide (PI) are nuclear DNA staining (nucleic acid binding) dyes. AO is permeable to both live and dead cells and stains all nucleated cells, generating green fluorescence. This assay was conducted to assess the morphology and quantify the viable and nonviable (apoptotic) cells. Viable cells are indicated by green nuclei with round intact structure and nonviable cells will display orange-to-red areas.

NHPL fibroblast cells were quantified using AOPI staining, according to standard procedures and examined under a fluorescence microscope (Lieca attached with Q-Floro Software, Solms, Germany). The treatment was carried out in a 25 mL culture flask (Nunc, Roskilde, Denmark). NHPL fibroblast cells were cultured at a concentration of 2 × 10^5^ cell mL^−1^ and treated with different MWs of chitosan (LMW, MMW and HMW) combined with a 0.024 μg mL^−1^ concentration of OPG. Flasks were incubated in an atmosphere of 5% CO_2_ at 37 °C for 24, 48 and 72 h. The cells were then spun down at 220 g for 5 min. The supernatant was discarded and the cells were washed twice, using cold PBS after being centrifuged at 220 g for 5 min to remove the remaining media. Five microliters of fluorescent dye containing AO (10 μg mL^−1^) and PI (10 μg mL^−1^) were added into the cellular pellet at equal volumes. The freshly stained cell suspension was dropped onto a glass slide and covered with a cover slip. The slides were then observed under the fluorescence microscope within 30 min before the fluorescent colour begin to fade. The percentages of viable and nonviable cells were determined based on the morphological criteria assessed under the UV-fluorescence microscope.

### 3D cell encapsulation in cell culture plates

In 96 well plates, BD PuraMatrix/cell/sucrose mixture was prepared according the protocol to the center of the well carefully, without introducing bubbles (Abu-Yousif et al., 2009). The cells were seeded into 96-well plate at a density of 10 × 10^3^ per well. After the cells have been plated in all wells, gelation of the PuraMatrix was initiated by gently running culture media down the side of the well on top of the hydrogel. The media was changed gently two times over the next one hour to further equilibrate the pH of the hydrogel. The treatment was started at 7days post cell seeding and exposed for 24, 48 and 72 h. At the end of the treatment, cell viability was estimated by AlamarBlue assay (ThermoFisher Scientific, Hempstead, UK).

### Western blot

Normal human osteoblasts were incubated in osteogenic medium at controlled conditions (5% CO2, 95% air and 37 °C). Osteoblast of second passage was used in this experiment. 75 mL cell culture flasks were used to seed the control and the cells were treated with OPG (0.024 μg mL^−1^ and LM chitosan (100 μg mL^−1^). Subsequently, these cells were incubated for 24, 48 and 72 h. PRO-PREPTM (iNtRON, Biotechnology, Korea) was used to extract the whole proteins, and NanoOrange protein quantitation kit (Invitrogen) was employed for protein quantification. The proteins were transformed with nitrocellulose paper. The nitrocellulose blot was blocked with a solution of 4% (W/V) dry milk for 3 h. The blot was incubated with monoclonal osteopontin (clone 1B20; Novus Biologicals, Cambridge, UK) and osteocalcin (OCG3; Abcam, Cambridge, UK) at a 1:1000 dilution for overnight. The goat anti-mouse secondary antibody was added at dilution of 1:5000 for 3 h. After the final wash, the proteins bands were detected on the membrane with calorimetric horseradish peroxidase (HRP) substrate, 4-chloro-1-naphthol (4CN) (Bio-Rad) kit. To capture images of the membrane, a UV gel documentation system (Biospectrum 410; UVP) was used.

### Real-time PCR analysis

The NHPL fibroblast cells were seeded and cultured as described earlier, then the cells were treated with OPG (0.024 μg mL^−1^ and LM chitosan (100 μg mL^−1^). Subsequently, these cells were incubated for 24, 48 and 72 h. RNA was extracted by RNeasy Mini Kit (Qiagen, Valencia, CA, USA). The concentration and purity of RNA were measured by using the NanoDrop 2000 spectrophotometer (Thermo Fisher Scientific, Waltham, MA, USA). cDNA was synthesized from the RNA following the manufacturer’s instructions using High Capacity RNA-to-cDNA Kit for RT-PCR (Applied Biosystems, Foster City, CA, USA). For the PCR reaction, TaqMan^®^Fast Advanced Master Mix was used (ThermoFisher Scientific, Hempstead, UK). The primers (for caspase 8, BCL2) that were used in this experiment. Glyceraldehyde phosphate dehydrogenase (GAPDH), a housekeeping gene, was used as an internal control. All of the assays were conducted in 96-well PCR plates using the StepOne Plus Real-Time PCR system (Applied Biosystems Inc, Waltham, MA, USA). The reactions were performed in triplicate. The mRNA level of each gene relative to that of GAPDH was calculated using the comparative quantification method

### Statistical analysis

For each microplate, reading values calculated from the exposed cells were converted into percentages with the negative control values considered to be 100%. The data was reported as the mean ± standard deviation (SD).

## Result

### The cell viability after treated with different MW chitosan

Table 1 summarises and compares the percentage of viable NHPL fibroblasts following treatment with different MWs of chitosan (LMW, MMW and HMW) over different time exposures and under control conditions using the MTT assay. Regardless of chitosan MW, the viability of the NHPL cells was ≥90%, following 24, 48 and 72 h of exposure as compared to the untreated cells.

**Table 1:** The percentage of NHPL fibroblast cells viability treated with different MWs of chitosan.

Time exposure (h)	Percentage of cell viability		
	LMW of chitosan 0.1 mg/mL	MMW of chitosan 0.1 mg/mL	HMW of chitosan 0.1 mg/mL
24	≥100%	≥92%	≥90%
48	≥100%	≥100%	≥96%
72	≥100%	≥100%	≥100%

**Notes.**

The viability percentage values were obtained from the MTT assay.

### The viability assay of OPG

**Figure 1:**
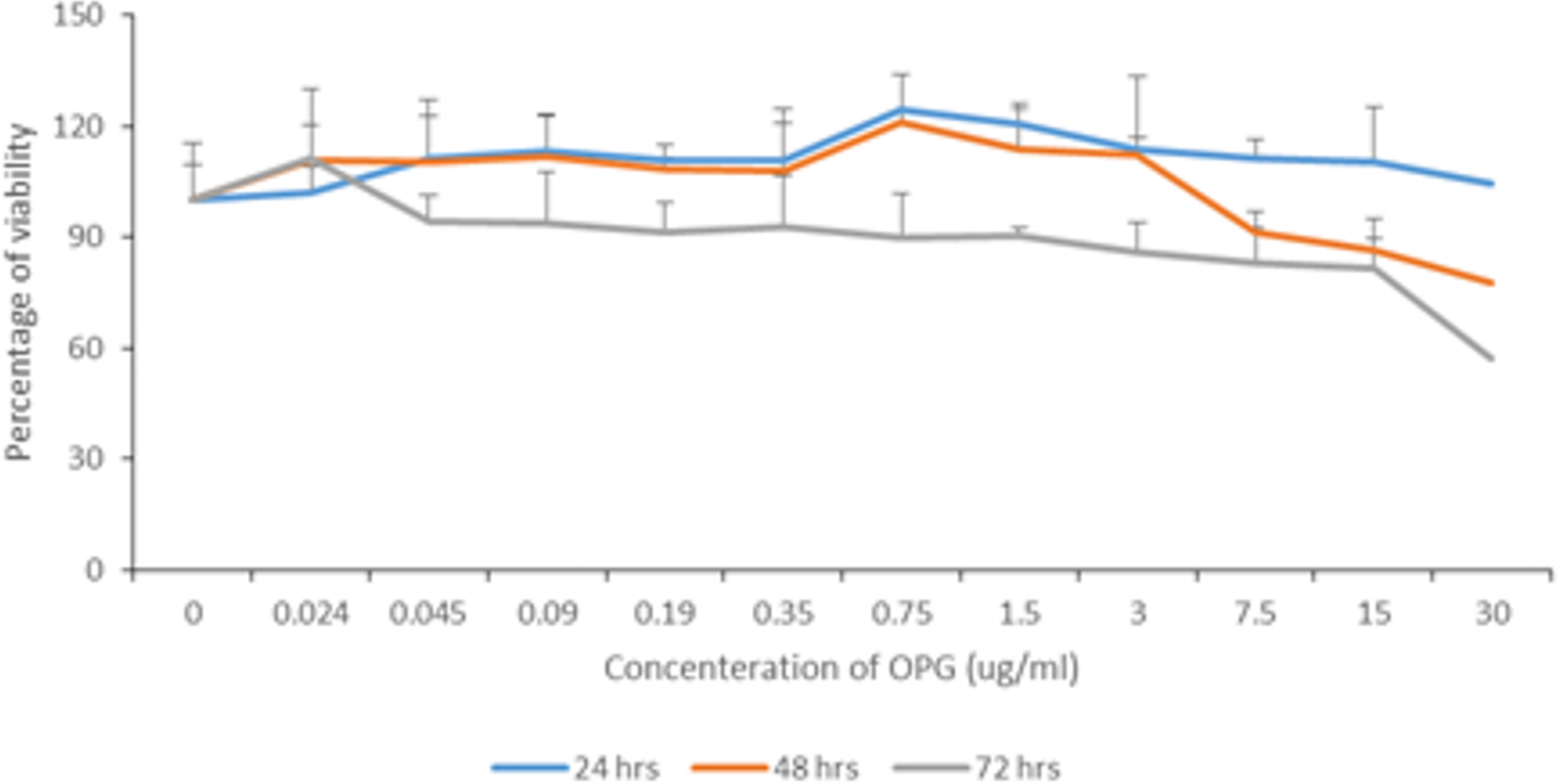
Percentage of NHPL fibroblast cells viability after treatment with different OPG doses (0–30 µg/mL) after 24, 48 and 72 h.

Figure 1 summarises the percentages of viability according to different OPG concentrations at different exposure times. In general, the viability of cells decreased gradually as the OPG concentration and exposure time increased. At 30 μg mL^−1^, the viability was reduced to less than 80% after 48 h and to less than 60% after 72 h. The viability of cells treated with 0.024–1.5 μg mL^−1^ OPG was ≥90% after 72 h of exposure.

### Proliferation assay of OPG

**Figure 2:**
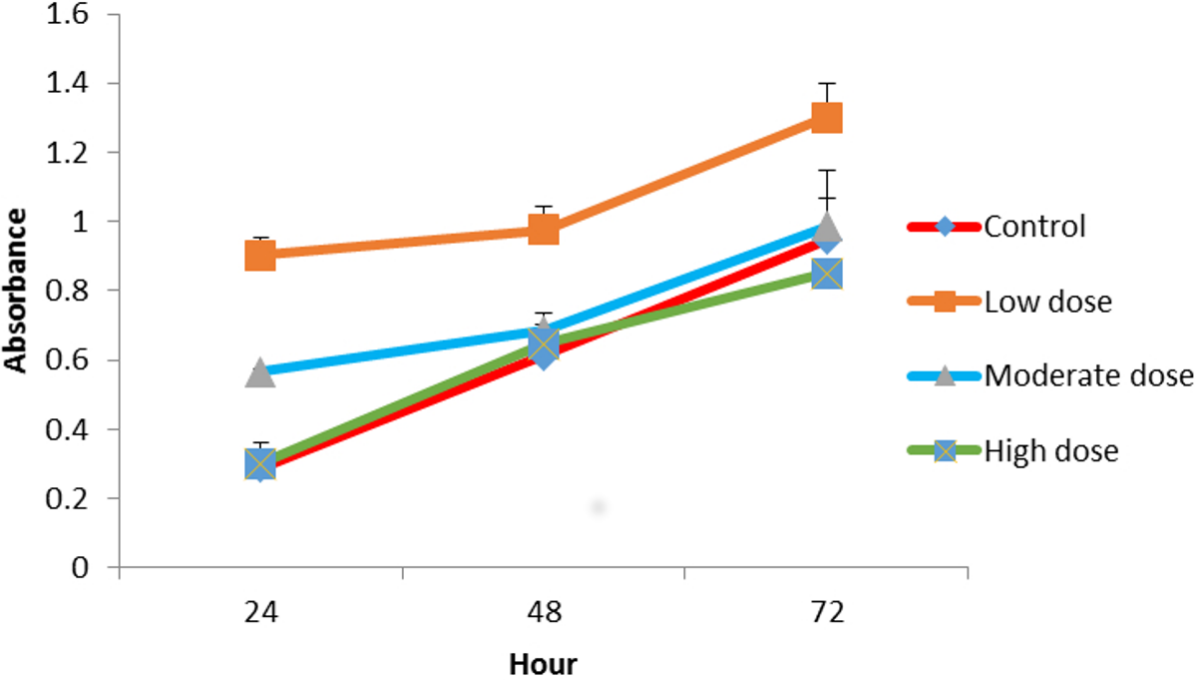
Comparison of cell proliferation rates at control, low (0.024 µg mL^−1^), moderate (0.15 µg mL^−1^) and high (1.5 µg mL^−1^) doses of OPG compared to control.

The effect of OPG on cell proliferation was studied *in vitro*. The cells treated with OPG showed optical densities for two concentration levels (0.024 μg mL^ −1^ and 0.18 μg mL^−1^) that were higher than cells treated with 1.5 μg mL^−1^ OPG and the controls (zero concentration OPG). Figure 2 shows the growth rates of the treated cells compared to the untreated cells. The highest cell proliferation rate was displayed at the concentration of 0.024 μg mL^−1^ after 72 h of incubation.

### Proliferation assay of LMW chitosan combined with different concentrations of OPG

**Figure 3:**
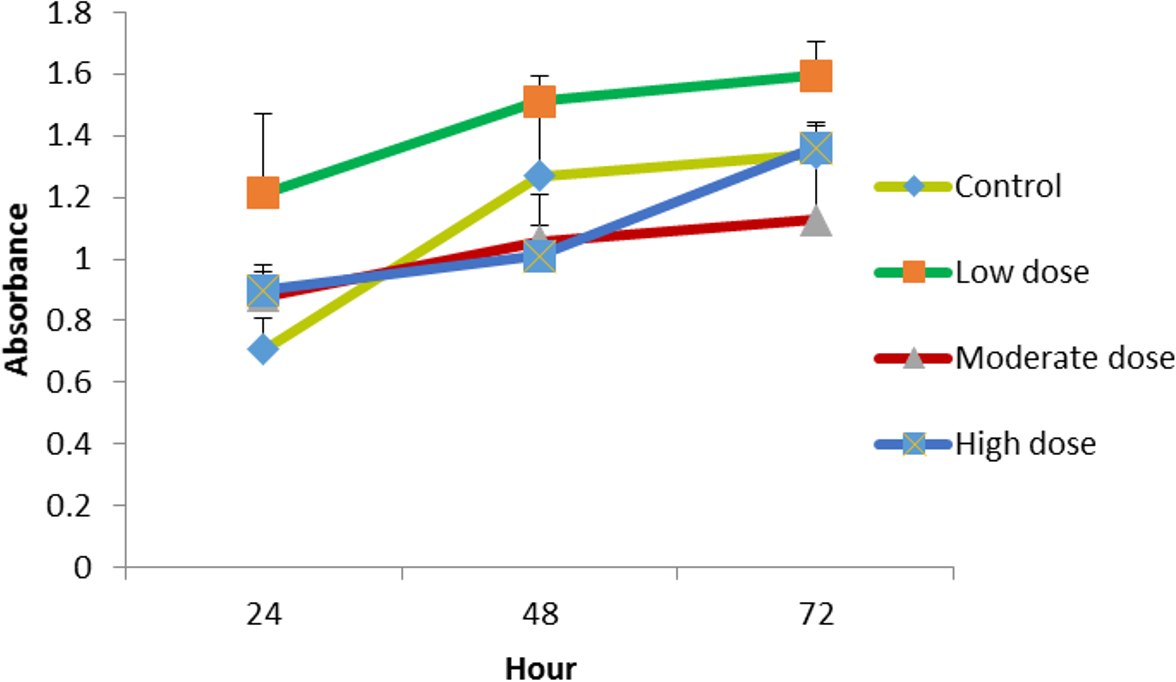
The effect of LMW chitosan combined with low (0.024 µg mL^−1^), moderate (0.15 µg mL^−1^) or high (1.5 µg mL^−1^) doses of OPG on NHPL fibroblasts proliferation *in-vitro*. NHPL Fibroblasts were treated for 24, 48 and 72 h.

When combined with 0.024 μg mL^−1^ OPG the LMW chitosan showed a higher cell proliferation rate than LMW chitosan combined with 1.5 and 0.18 μg mL^−1^ concentrations of OPG (Fig. 3).

### Proliferation assay of MMW chitosan combined with different concentrations of OPG

**Figure 4:**
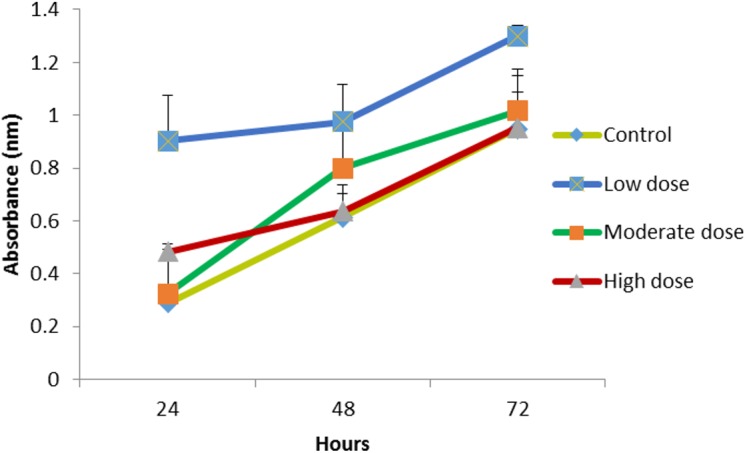
The effect of MMW chitosan combined with OPG (low (0.024 µg mL^ −1^), moderate (0.15 µg mL^−1^) or high (1.5 µg mL^−1^) dose) on NHPL fibroblasts proliferation *in-vitro*. NHPL fibroblast cells were treated for 24, 48 and 72 h.

The cells were treated with MMW chitosan (100 μg mL^−1^ combined with different concentration of OPG (1.5, 0.18, 0.024 μg mL^−1^), the proliferation of cells was evaluated at 24, 48, 72 h. Chitosan combined with 0.024 μg mL^ −1^ OPG showed a higher cell proliferation rate than chitosan combined with 1.5, 0.18 μg mL^−1^ OPG over three different exposure time points (Fig. 4).

### Proliferation assay of HMW chitosan combined with different concentrations of OPG

**Figure 5:**
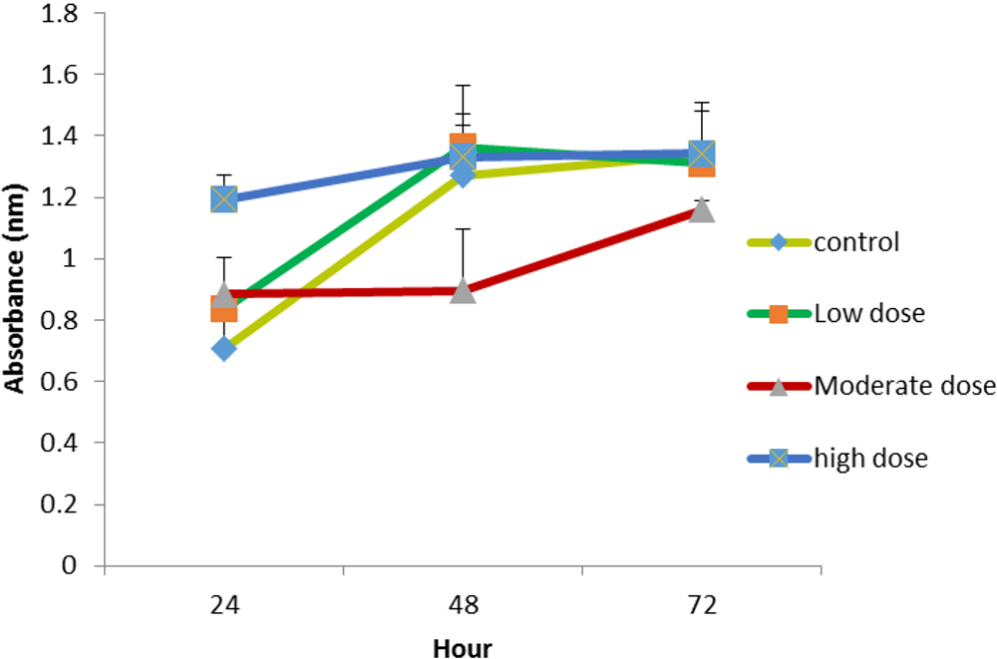
The effect of HMW chitosan samples in different concentrations of OPG (low (0.024 µg mL^−1^), moderate (0.15 µg mL^ −1^) or high (1.5 µg mL^−1^) dose) on NHPL fibroblasts proliferation *in-vitro*. NHPL fibroblast cells were treated for 24, 48 and 72 h.

The HMW chitosan combined with the same concentrations of OPG (0.024, 0.18 and 1.5 μg mL^−1^) were used to evaluate the growth of the cells. The 1.5 μg mL^−1^ OPG-chitosan combination induced a greater proliferation of cells than the other two combinations after 24 h. However, there was no marked difference in the proliferation of cells at 0.024 μg mL^−1^ and 1.5 μg mL^−1^ concentration levels after 72 h of incubation, and they showed an increase of cell proliferation, when compared to the control (Fig. 5).

### Proliferation assay of three different MWs of chitosan combined with 0.024 µg/mL OPG concentration using 3D culture system

**Figure 6:**
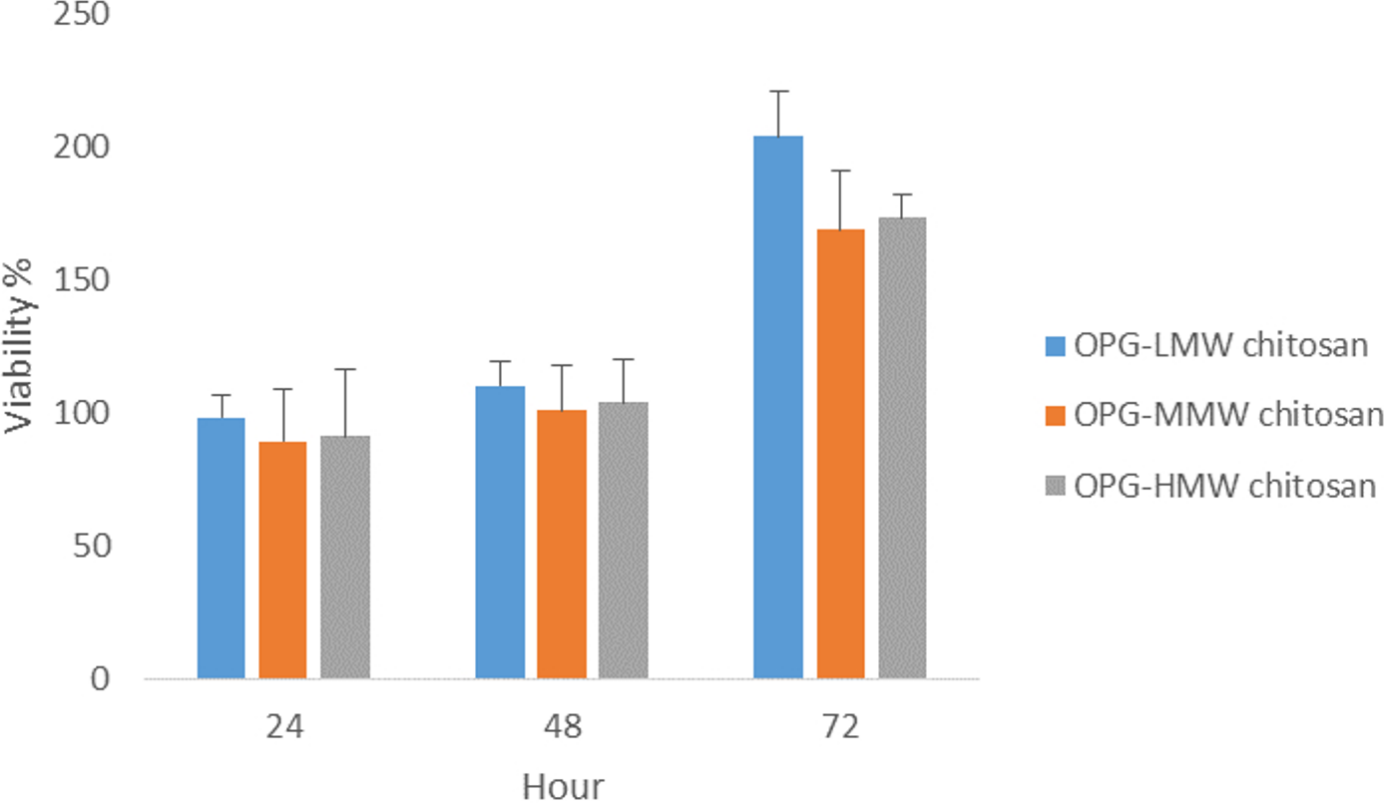
The effect of HMW, MMW and LMW chitosan combined with 0.024 µg/mL of OPG on NHPL fibroblasts proliferation *in-vitro*. NHPL fibroblast cells were treated for 24, 48 and 72 h.

Previous results of the viability and proliferation assays (Figs. 3–5) showed 0.024 μg mL^−1^ concentration of OPG is the optimum concentration to use. It has been shown to be nontoxic and enhance the proliferation of cells. Another proliferation assay was carried out to determine the appropriate MW of chitosan (100 μg mL^−1^) to be used in combination with the 0.024 μg/mL OPG. The result revealed that LMW chitosan in combination with the 0.024 μg/mL OPG demonstrated higher cell proliferation, compared to MMW and HMW chitosan (Fig. 6).

### Morphological changes of cells after treatment with OPG-chitosan combinations

**Figure 7:**
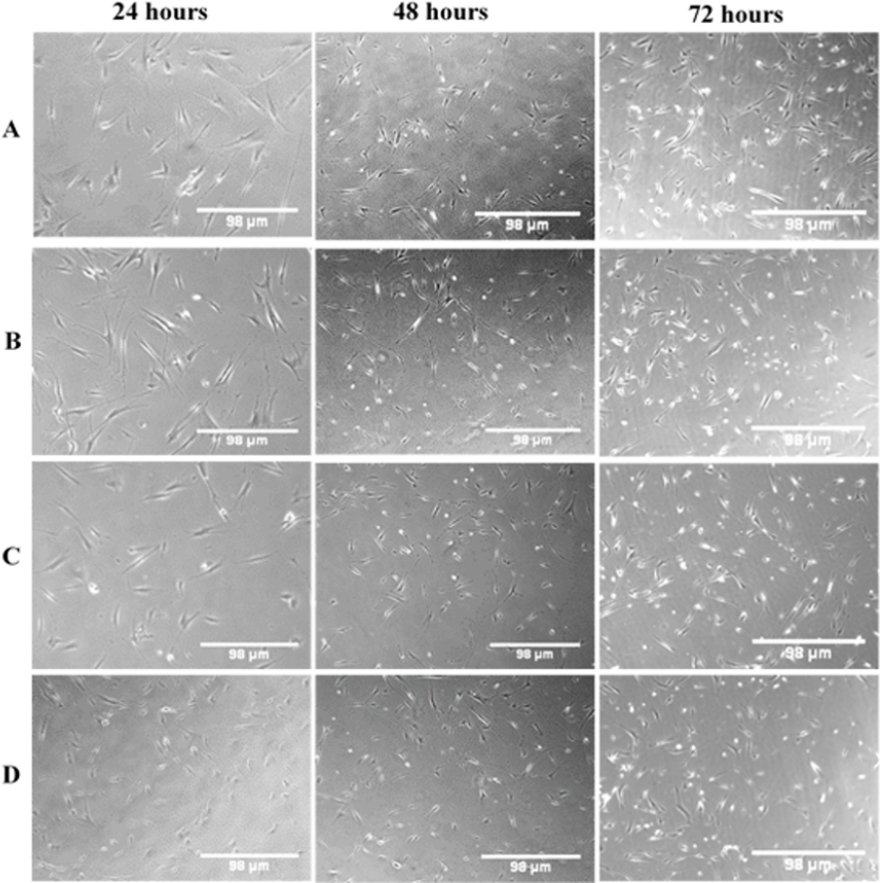
The morphological changes of NHPL fibroblast cells observed under an inverted microscope after 24, 48 and 72 h. (A) Control (untreated cells), (B) OPG-LMW chitosan combination, (C) OPG-MMW chitosan combination and (D) OPG-HMW chitosan combination.

In general, regardless of the MW of chitosan, the treated cells appeared to have normal morphology, such as flattened surfaces, and they adhered to the surface of well with very minimal rounded cells (not attached cells) at the various exposure times. Figure 7 is a phase contrast image showing morphological changes of NHPL fibroblast cells, following treatment with LMW, MMW and HMW chitosan in combination with 0.024 μg/mL concentration of OPG. We noticed that there was no difference between the morphology of cells treated with gels compared to the control group of untreated cells. We suggest that OPG had no effect on the morphology of the cells.

### Quantification of the cell viability using AOPI double-staining

**Figure 8:**
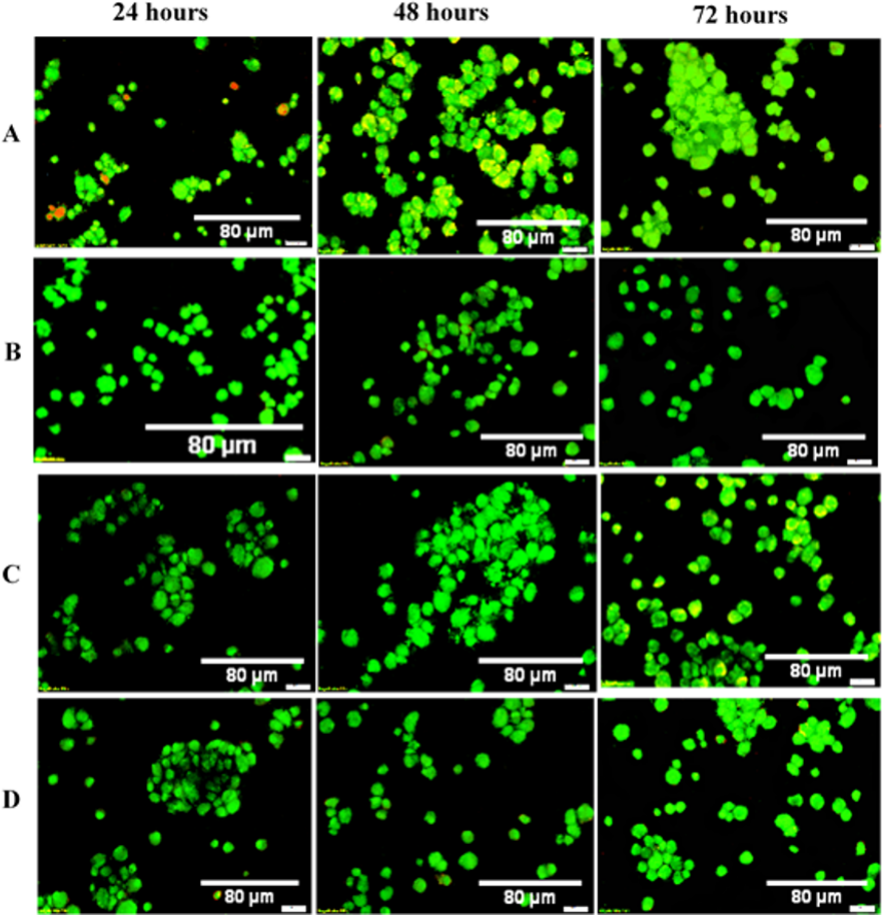
AOPI Viability: dual fluorescence for viable and nonviable cells treated with OPG-chitosan combinations and untreated cells. Images were observed at 50× magnification. (A) Control (untreated cells), (B) OPG-LMW chitosan combination, (C) OPG-MMW chitosan combination and (D) OPG-HMW chitosan combination.

In general, following exposure to different OPG-chitosan combinations at different time exposures, most of the cells were viable and showed fluorescent green with intact cell walls and minimal amounts of dead cells. Figure 8 shows the fluorescent images of the NHPL fibroblast cells, following treatment with LMW, MMW and HMW chitosan in combination with 0.024 μg/mL OPG after 24, 48 and 72 h. Table 2 shows cell viability percentages after they were treated with OPG in combination with different MWs of chitosan. The cell viability percentages ranged from 87% to 92%, 89% to 92% and 89% to 92% after 24, 48 and 72 h respectively

### Osteopontin and osteocalcin protein levels

**Figure 9:**
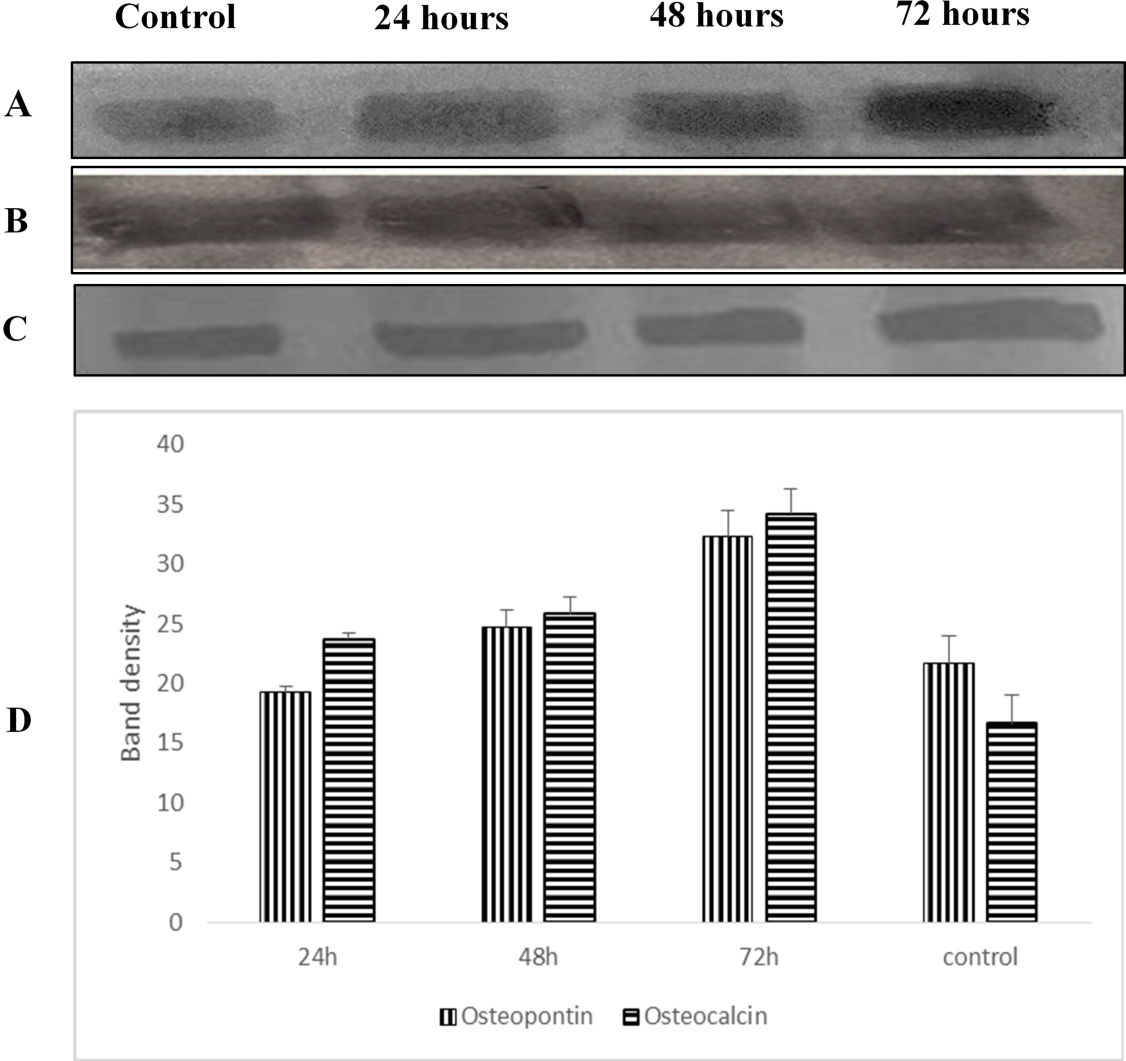
Effect of LMW chitosan combined with 0.024 μg/mL of OPG on osteopontin (A) and osteocalcin (C) proteins expression at 24, 48 and 72 hours. GAPDH (B) was used as a loading control. (D) Quantitative analysis of treatment. All data were expressed as means ± standard deviation (SD).

Western blot analysis was used to examine the expression level of osteopontin and osteocalcin protein in treated normal human osteoblast cells with LMW chitosan combined with 0.024 μg mL^−1^ of OPG. Results in Fig. 9 revealed that LMW chitosan combined with 0.024 μg mL^−1^ of OPG induce the expression levels of bone formation marker proteins osteopontin and osteocalcin in time-dependent manner. At 24 h post treatment, proteins level were lower and increased after 48 and 72 h treatment.

### mRNA expression of caspase 8 and BCL2

**Figure 10:**
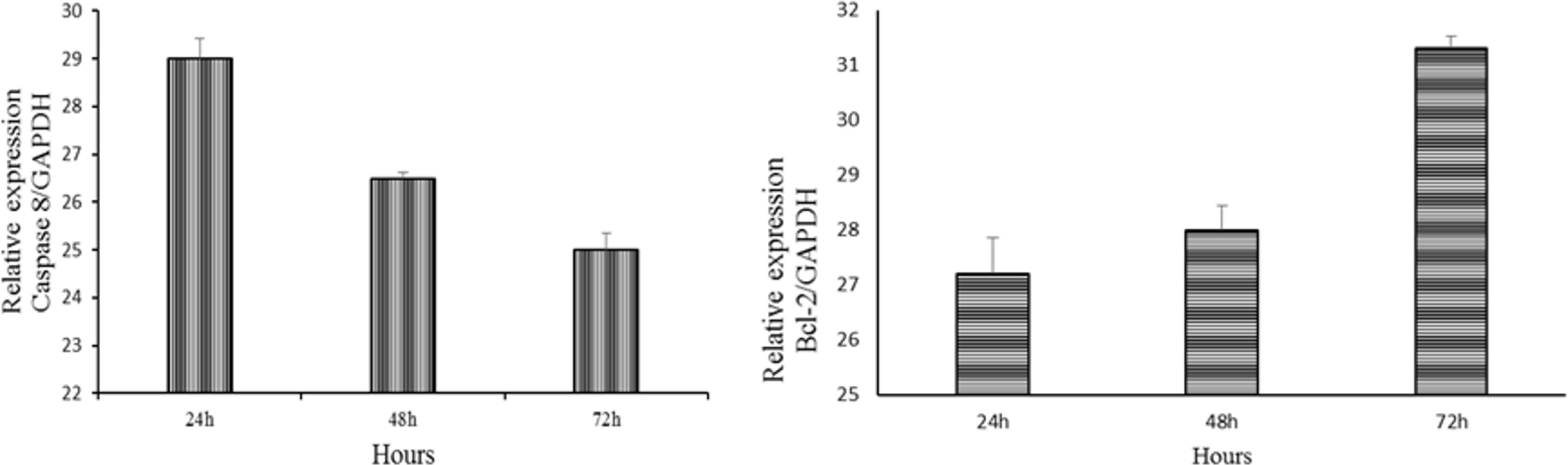
Downregulation of caspase 8 and upregulation of Bcl-2 expression in NHPL fibroblast cells. The expression of caspase 8 and Bcl-2 after 24, 48 and 72 h of treatment was studied by RT-PCR. GAPDH was used as internal (positive).

A real-time PCR analysis was performed to investigate the differences in the mRNA expression levels of caspase 8 and Bcl-2 which are apoptosis-related genes. The results showed the downregulation of caspase 8 and upregulation of Bcl-2 expression after treatment with LMW chitosan combined with 0.024 μg mL^−1^ of OPG (Fig. 10).

**Table 2:** The cell viability percentages treated with OPG combined with different MW chitosan.

Combinations	24 (h)	48 (h)	72 (h)
Control (untreated cells)	87 ± 10%	89 ± 13%	92 ± 14%
OPG in LMW chitosan	91 ± 16%	85 ± 15%	89 ± 20%
OPG in MMW chitosan	92 ± 19%	92 ± 18%	90 ± 11%
OPG in HMW chitosan	88 ± 12%	90 ± 9%	91 ± 7%

**Notes.**

The viability percentages of different MW chitosan were combined with OPG on NHPL cell lines *in vitro* at 24, 48 and 72-hour treatments. The viability percentage values were obtained from the AOPI double-staining assay. Data are reported as means ± SD for measurements in triplicate.

## Discussion

Different MWs of chitosan showed no marked inhibition on the viability of the NHPL fibroblast cells. This study also confirmed that chitosan has the ability to induce proliferation of NHPL fibroblast cells, which serve a role in bone healing. We used NHPL fibroblasts in this study as Fibroblasts are one of the important cells involved in the healing process. The NHPL fibroblast cell is one the main cells that contribute in the periodontal bone regeneration as it has osteoblast-like properties such as alkaline phosphatase activity, vitamin D-dependent production of osteocalcin and initiation of mineral-like nodules in the presence of a supportive medium (Jönsson et al., 2011; Scanlon et al., 2011; Basdra & Komposch, 1997). The extent of chitosan’s ability to regenerate bone is still debated. Spin-Neto and co-workers (Spin-Neto et al., 2012; Spin-Neto et al., 2010) revealed that there was no significant bone formation following chitosan and chitosan hydrochloride gel application in critical sizes of bone defects; the defects were repaired by connective tissue with variable degrees of inflammation. On the other hand, Muzzarelli et al. (1994) reported that chitosan enhanced osseous healing of defects created in sheep. Jung et al. (2000) also reported that chitosan has significant effects on the regeneration of bone tissue in calvarial defects in rats. Other studies have reported that chitosan and chitosan-based biomaterials, tested in the treatment of bone defects, have a high degree of biocompatibility, osteoconductivity and increased the density of newly formed bone (Bojar et al., 2014; Florczyk et al., 2013; Jung et al., 2013; Lee et al., 2000), and chitosan derivatives have the ideal properties of biocompatible materials tested on normal human fibroblasts (Spin-Neto et al., 2012; Spin-Neto et al., 2010; Muzzarelli et al., 1994). Biocompatibility is one of the most important criteria in selecting biomaterials. Clinically, chitosan also has high potential in dental applications, and it has used to repair socket after dental extraction (Ezoddini-Ardakani, 2011). Other research also reported that biodegradable dental chip containing chlorhexidine or thymoquinone was applied for management of chronic periodontitis in patients (Al-Bayaty et al., 2013; Jothi et al., 2009).

With regards to OPG, the viability of NHPL fibroblast cells was significantly high at concentrations of 0.024 and up to 3 μg mL^−1^ of OPG. Previous studies have reported that OPG acts as a survival factor, at least *in vitro*, by blocking TRAIL-induced apoptosis (Lane et al., 2012; Holen et al., 2002). Several preclinical studies that used OPG systemically for treatment of bone disorders have revealed that OPG inhibits bone resorption and improves osteoblastogenesis and new bone formation (Yao et al., 2011; Jin et al., 2007; Lamoureux et al., 2007). Other studies involving clinical use of OPG to treat bone loss in post-menopausal women revealed that biochemical markers of bone resorption were reduced and OPG was able to lessen the amount of bone turnover (Bekker et al., 1999). Body and co-workers (Body et al., 2003) stated that OPG was accepted as a treatment for patients with bone disease related to breast carcinoma or multiple myeloma and is thus effective in reducing levels of bone resorption markers. This is the first study to report on the toxicity evaluation of OPG in cells. The results demonstrated that a low concentration of OPG (0.024 mg mL^−1^) has the greatest ability to induce proliferation of NHPL fibroblast cells compared to other concentrations. On the other hand, the proliferation of the cells was greater when OPG (0.024 mg mL^−1^) was combined with chitosan (low and moderate molecular weights) compared to separate treatments with chitosan and OPG; but the combination with high molecular weight chitosan exhibited no difference among three doses of OPG.

3D cell culture models produce a pragmatic microenvironment and simulate an *in vivo* system, which aids to understand cell–cell interactions (Yamada & Cukierman, 2007). Cells cultured in a 3D environment have the ability to acquire phenotypes and respond to stimuli similar to *in vivo* biological systems (Prestwich, 2007; Godugu et al., 2013). This study showed that the LMW chitosan combined with OPG had the greatest ability to induce cells proliferation compared to the moderate and high molecular weights of chitosan combined with OPG. This is in accordance with Chen et al. (2002) and Nor Asiah et al. (2013) who have reported that low molecular weight chitosan significantly promoted growth of normal fibroblasts. Other studies also reported that fibroblasts treated with low molecular weight chitosan stimulated fibroblasts proliferation compared to chitosan at higher molecular weights (Tangsadthakun et al., 2007; Wang et al., 2007).

The results also verified that the rate of NHPL fibroblast cells proliferation increased with time exposure to the OPG-chitosan matrixes and did not cause toxicity effects on fibroblast cell growth. This is also in agreement with a study reported by Nor Asiah et al. (2013).

Regarding the effect of on the activity of NH osteoblast cells, human osteoblasts were treated with LMW chitosan combined with OPG with optimal concentration confirmed by the proliferation assays. The results showed that the osteocalcin and osteopontin levels were increased as time of exposure increased. Based on these findings, we may postulate this treatment has ability to enhance the differentiation of cells as Celic and coworkers have demonstrated that the production of some bone protein such as osteocalcin was increased in differentiated cells as it is late marker of bone formation (Celic et al., 1998). Osteocalcin is a mineralization-specific marker because its expression increases as the mineralization increase (Yamada et al., 2013). Xiao et al. (2004) reported that there is a correlation between calcification and the distribution of bone sialoprotein and osteopontin.

OPG seems also play a key role in cell survival, via its interaction with TNF-related apoptosis-inducing ligand (TRAIL). OPG can act as a decoy receptor for TNF-related apoptosis inducing ligand, because it is efficiently binds with TRIAL. TRAIL signaling leads to cell death by activation of caspase-8 leads to caspase cascade that culminate in cell death (Baud’huin et al., 2013; Crowder & El-Deiry, 2012). Also, overexpression of Bcl-2 was found to inhibit the TRIAL-induced caspase 8, thus inhibiting the TRAIL-induced apoptosis in many cells. The results of RT-PCR revealed that the downregulation of caspase 8 and upregulation of Bcl-2 that may promote cell proliferation. These are in agreement with previous studies that reported the exogenous application of recombinant OPG has indeed been shown to be capable of inhibiting TRAIL-induced apoptosis and subsequently downregulate the caspase 8 expression (Lemke et al., 2014; Miyashita et al., 2004; Shipman & Croucher, 2003).

The LMW chitosan has structural characteristics similar to those of the glycosaminoglycans that facilitate the migration and proliferation of cells and the also the OPG has effects on the survival of cells, thereby the OPG-chitosan combination facilitate the tissue regeneration.

This is the first study that evaluated the combination effect of OPG with different molecular weights of chitosan on osteoblast and NHPL fibroblast. The results of this study indicate the LMW chitosan combined with OPG has potential to be used as a biomaterial for bone tissue engineering.

## Conclusion

Our results have suggested OPG in low molecular weight chitosan matrixes enhances cell growth and proliferation, and induce the production of osteopontin and osteocalcin protein levels. It can be used in different local preparations for potential bone defect application. Further study is necessary to clarify the effect of combining OPG and chitosan for bone management applications.

## Supplemental Information

10.7717/peerj.2229/supp-1 Table S1 The viability assay of OPG

10.7717/peerj.2229/supp-2 Table S2 Proliferation assay of OPG

10.7717/peerj.2229/supp-3 Table S3 Proliferation assay of LMW chitosan combined with different concentrations of OPG

10.7717/peerj.2229/supp-4 Table S4 Proliferation assay of LMW chitosan combined with different concentrations of OPG

10.7717/peerj.2229/supp-5 Table S5 Proliferation assay of HMW chitosan combined with different concentrations of OPG

10.7717/peerj.2229/supp-6 Table S6 Proliferation assay of three different MWs of chitosan combined with 0.024 µg/mL OPG concentration using 3D culture system

10.7717/peerj.2229/supp-7 Table S7 Osteopontin and osteocalcin protein levels

## Additional Information and Declarations

### Data Deposition

The following information was supplied regarding data availability: The raw data was supplied as Supplemental Dataset.

## References

[ref-1] Abu-Yousif AO, Rizvi I, Evans CL, Celli JP, Hasan T (2009). PuraMatrix encapsulation of cancer cells. Journal of Visualized Experiments.

[ref-2] Al-Bayaty FH, Kamaruddin AA, Ismail MA, Abdulla MA (2013). Formulation and evaluation of a new biodegradable periodontal chip containing thymoquinone in a chitosan base for the management of chronic periodontitis. Journal of Nanomaterials.

[ref-3] Baharuddin N, Coates D, Cullinan M, Seymour G, Duncan W (2015). Localization of RANK, RANKL and osteoprotegerin during healing of surgically created periodontal defects in sheep. Journal of Periodontal Research.

[ref-4] Basdra EK, Komposch G (1997). Osteoblast-like properties of human periodontal ligament cells: an *in vitro* analysis. The European Journal of Orthodontics.

[ref-5] Baud’huin M, Duplomb L, Teletchea S, Lamoureux F, Ruiz-Velasco C, Maillasson M, Redini F, Heymann MF, Heymann D (2013). Osteoprotegerin: multiple partners for multiple functions. Cytokine and Growth Factor Reviews.

[ref-6] Bekker PJ, Holloway D, Nakanishi A, Arrighi M, Leese PT, Dunstan CR (2001). The effect of a single dose of osteoprotegerin in postmenopausal women. Journal of Bone and Mineral Research.

[ref-7] Bekker PJHD, Nakanishi A, Arrighi HM, Dunstan CR (1999). Osteoprotegerin (OPG) has potent and sustained anti-resorptive activity in postmenopausal women. Journal of Bone and Mineral Research.

[ref-8] Body JJ, Greipp P, Coleman RE, Facon T, Geurs F, Fermand JP, Harousseau JL, Lipton A, Mariette X, Williams CD (2003). A phase I study of AMGN-0007, a recombinant osteoprotegerin construct, in patients with multiple myeloma or breast carcinoma related bone metastases. Cancer.

[ref-9] Bojar W, Kucharska M, Ciach T, Koperski Ł, Jastrzębski Z, Szałwiński MS (2014). Bone regeneration potential of the new chitosan-based alloplastic biomaterial. Journal of Biomaterials Applications.

[ref-10] Bostanci N, ilgenli T, Emingil G, Afacan B, Han B, Töz H, Atilla G, Hughes FJ, Belibasakis GN (2007). Gingival crevicular fluid levels of RANKL and OPG in periodontal diseases: implications of their relative ratio. Journal of Clinical Periodontology.

[ref-11] Celic S, Katayama Y, Chilco P, Martin T, Findlay D (1998). Type I collagen influence on gene expression in UMR106-06 osteoblast-like cells is inhibited by genistein. Journal of Endocrinology.

[ref-12] Chen X-G, Wang Z, Liu W-S, Park H-J (2002). The effect of carboxymethyl-chitosan on proliferation and collagen secretion of normal and keloid skin fibroblasts. Biomaterials.

[ref-13] Coimbra P, Alves P, Valente T, Santos R, Correia I, Ferreira P (2011). Sodium hyaluronate/chitosan polyelectrolyte complex scaffolds for dental pulp regeneration: synthesis and characterization. International Journal of Biological Macromolecules.

[ref-14] Crowder RN, El-Deiry WS (2012). Caspase-8 regulation of TRAIL-mediated cell death. Experimental Oncology.

[ref-15] Ezoddini-Ardakani F (2011). Effects of chitosan on dental bone repair. Health.

[ref-16] Florczyk SJ, Leung M, Li Z, Huang JI, Hopper RA, Zhang M (2013). Evaluation of three-dimensional porous chitosan–alginate scaffolds in rat calvarial defects for bone regeneration applications. Journal of Biomedical Materials Research Part A.

[ref-17] Godugu C, Patel AR, Desai U, Andey T, Sams A, Singh M (2013). AlgiMatrix™ based 3D cell culture system as an *in-vitro* tumor model for anticancer studies. PLoS ONE.

[ref-18] Hofbauer LC, Khosla S, Dunstan CR, Lacey DL, Spelsberg TC, Riggs BL (1999). Estrogen stimulates gene expression and protein production of osteoprotegerin in human osteoblastic cells*. Endocrinology.

[ref-19] Holen I, Croucher PI, Hamdy FC, Eaton CL (2002). Osteoprotegerin (OPG) is a survival factor for human prostate cancer cells. Cancer Research.

[ref-20] Ilium L (1998). Chitosan and its use as a pharmaceutical excipient. Pharmaceutical Research.

[ref-21] Jin Q, Cirelli JA, Park CH, Sugai JV, Taba Jr M, Kostenuik PJ, Giannobile WV (2007). RANKL inhibition through osteoprotegerin blocks bone loss in experimental periodontitis. Journal of Periodontology.

[ref-22] Jönsson D, Nebel D, Bratthall G, Nilsson BO (2011). The human periodontal ligament cell: a fibroblast-like cell acting as an immune cell. Journal of Periodontal Research.

[ref-23] Joshi DP, Mehta NK, Shah JS, Shah VH (2012). Chitosan nanospheres as potential carrier delivery of pharmaceutical API¡-s. International Journal of Pharmaceutical and Phytopharmacological Research.

[ref-24] Jothi M, Bhat K, Pratibha P, Bhat G (2009). The evaluation of a biodegradable dental chip containing chlorhexidine in chitosan base as a targeted drug delivery in the management of chronic periodontitis in patients. Drug Development Research.

[ref-25] Jung UW, Suh JJ, Choi SH, Cho KS, Chai JK, Kim CK (2000). The bone regenerative effects of chitosan on the calvarial critical size defectin sprague dawley rats. The Journal of the Korean Academy of Periodontology.

[ref-26] Jung HD, Yook SW, Han CM, Jang TS, Kim HE, Koh YH, Estrin Y (2013). Highly aligned porous Ti scaffold coated with bone morphogenetic protein-loaded silica/chitosan hybrid for enhanced bone regeneration. Journal of Biomedical Materials Research Part B: Applied Biomaterials.

[ref-27] Koide M, Kobayashi Y, Ninomiya T, Nakamura M, Yasuda H, Arai Y, Okahashi N, Yoshinari N, Takahashi N, Udagawa N (2013). Osteoprotegerin-deficient male mice as a model for severe alveolar bone loss: comparison with RANKL-overexpressing transgenic male mice. Endocrinology.

[ref-28] Kostenuik PJ, Capparelli C, Morony S, Adamu S, Shimamoto G, Shen V, Lacey DL, Dunstan CR (2001). OPG and PTH-(1–34) have additive effects on bone density and mechanical strength in osteopenic ovariectomized rats. Endocrinology.

[ref-29] Lamoureux F, Richard P, Wittrant Y, Battaglia S, Pilet P, Trichet V, Blanchard F, Gouin F, Pitard B, Heymann D (2007). Therapeutic relevance of osteoprotegerin gene therapy in osteosarcoma: blockade of the vicious cycle between tumor cell proliferation and bone resorption. Cancer Research.

[ref-30] Lane D, Matte I, Rancourt C, Piché A (2012). Osteoprotegerin (OPG) protects ovarian cancer cells from TRAIL-induced apoptosis but does not contribute to malignant ascites-mediated attenuation of TRAIL-induced apoptosis. Journal of Ovarian Research.

[ref-31] Lee Y-M, Park Y-J, Lee S-J, Ku Y, Han S-B, Klokkevold PR, Chung C-P (2000). The bone regenerative effect of platelet-derived growth factor-BB delivered with a chitosan/tricalcium phosphate sponge carrier. Journal of Periodontology.

[ref-32] Lemke JV, Von Karstedt S, Zinngrebe J, Walczak H (2014). Getting TRAIL back on track for cancer therapy. Cell Death and Differentiation.

[ref-33] Miyashita T, Kawakami A, Nakashima T, Yamasaki S, Tamai M, Tanaka F, Kamachi M, Ida H, Migita K, Origuchi T, Nakao K (2004). Osteoprotegerin (OPG) acts as an endogenous decoy receptor in tumour necrosis factor-related apoptosis-inducing ligand (TRAIL)-mediated apoptosis of fibroblast-like synovial cells. Clin Exp Immunol..

[ref-34] Muzzarelli R, Mattioli-Belmonte M, Tietz C, Biagini R, Ferioli G, Brunelli M, Fini M, Giardino R, Ilari P, Biagini G (1994). Stimulatory effect on bone formation exerted by a modified chitosan. Biomaterials.

[ref-35] Nagasawa T, Kobayashi H, Kiji M, Aramaki M, Mahanonda R, Kojima T, Murakami Y, Saito M, Morotome Y, Ishikawa I (2002). LPS-stimulated human gingival fibroblasts inhibit the differentiation of monocytes into osteoclasts through the production of osteoprotegerin. Clinical & Experimental Immunology.

[ref-36] Nor Asiah M, Halim AS, Shamsuddin S, Hussin CMC, Ujang Z, Rashid AHA (2013). The effect of chitosan derivatives film on the proliferation of human skin fibroblast: an-in vitro study. Journal of Sustainability Science and Management.

[ref-37] Prestwich GD (2007). Simplifying the extracellular matrix for 3-D cell culture and tissue engineering: a pragmatic approach. Journal of Cellular Biochemistry.

[ref-38] Scanlon CS, Marchesan JT, Soehren S, Matsuo M, Kapila YL (2011). Capturing the regenerative potential of periodontal ligament fibroblasts. Journal of Stem Cells and Regenerative Medicine.

[ref-39] Shipman CM, Croucher PI (2003). Osteoprotegerin is a soluble decoy receptor for tumor necrosis factor-related apoptosis-inducing ligand/Apo2 ligand and can function as a paracrine survival factor for human myeloma cells. Cancer Research.

[ref-40] Souza BDM, Lückemeyer DD, Felippe WT, Simōes CMO, Felippe MCS (2010). Effect of temperature and storage media on human periodontal ligament fibroblast viability. Dental Traumatology.

[ref-41] Spin-Neto R, Coletti FL, Freitas RMD, Pavone C, Campana-Filho SP, Marcantonio RAC (2012). Chitosan-based biomaterials used in critical-size bone defects: radiographic study in rat’s calvaria. Revista de Odontologia da UNESP.

[ref-42] Spin-Neto R, De Freitas RM, Pavone C, Cardoso MB, Campana-Filho SP, Marcantonio RAC, Marcantonio E (2010). Histological evaluation of chitosan-based biomaterials used for the correction of critical size defects in rat’s calvaria. Journal of Biomedical Materials Research Part A.

[ref-43] Tangsadthakun C, Kanokpanont S, Sanchavanakit N, Pichyangkura R, Banaprasert T, Tabata Y, Damrongsakkul S (2007). The influence of molecular weight of chitosan on the physical and biological properties of collagen/chitosan scaffolds. Journal of Biomaterials Science, Polymer Edition.

[ref-44] Wang Z, Liu W, Han B, Yao R, Wei C (2007). Preparation of carboxymethyl-chitosan with different molecular weight and its effects on proliferation of skin fibroblasts and keratinocytes. Sheng wu yi xue gong cheng xue za zhi = Journal of Bbiomedical Engineering = Shengwu yixue gongchengxue zazhi.

[ref-45] Xiao Y, Haase H, Young WG, Bartold M (2004). Development and transplantation of a mineralized matrix formed by osteoblasts *in vitro* for bone regeneration. Cell Transplantation.

[ref-46] Yamada KM, Cukierman E (2007). Modeling tissue morphogenesis and cancer in 3D. Cell.

[ref-47] Yamada S, Yoshizawa Y, Kawakubo A, Ikeda T, Yanagiguchi K, Hayashi Y (2013). Early gene and protein expression associated with osteoblast differentiation in response to fish collagen peptides powder. Dental Materials Journal.

[ref-48] Yao Y, Wang G, Wang Z, Wang C, Zhang H, Liu C (2011). Synergistic enhancement of new bone formation by recombinant human bone morphogenetic protein-2 and osteoprotegerin in trans-sutural distraction osteogenesis: a pilot study in dogs. Journal of Oral and Maxillofacial Surgery.

