# Supplementary material for: In vitro evaluation of osteoprotegerin in chitosan for potential bone defect applications
Source: PeerJ. 2016 Aug 23;4:e2229. doi: 10.7717/peerj.2229 (PMC5012333; doi:10.7717/peerj.2229)
Supplement: Table S1 [file peerj-04-2229-s001.docx]

|  | Viability percentages | | |  | standard deviation | | | |
| --- | --- | --- | --- | --- | --- | --- | --- | --- |
|  | 24 hrs | 48 hrs | 72 hrs |  |  | 24 hrs | 48 hrs | 72 hrs |
| control | 100 | 100 | 100 |  | control | 100 | 100 | 100 |
| 0.024 | 102.23 | 110.9466 | 111.4437 |  | 0.024 | 15.6 | 9.7 | 15.5 |
| 0.045 | 111.3 | 110.173 | 94.32966 |  | 0.045 | 7 | 19 | 9.2 |
| 0.09 | 113.2 | 111.9781 | 93.61461 |  | 0.09 | 11.5 | 17 | 7.2 |
| 0.19 | 111.01 | 108.3299 | 91.32258 |  | 0.19 | 10.3 | 11 | 14.3 |
| 0.35 | 110.7808 | 107.8358 | 92.86801 |  | 0.35 | 4.2 | 3.4 | 8.2 |
| 0.75 | 124.6634 | 120.965 | 90 |  | 0.75 | 10.1 | 17 | 13.8 |
| 1.5 | 120.4341 | 113.7863 | 90.22341 |  | 1.5 | 9.3 | 13.1 | 11.7 |
| 3 | 113.5955 | 112.136 | 85.76427 |  | 3 | 6 | 11.2 | 2.4 |
| 7.5 | 111.1648 | 91.2443 | 83.05841 |  | 7.5 | 20.2 | 4.7 | 8.1 |
| 15 | 110.6073 | 86.68017 | 81.72987 |  | 15 | 5.3 | 5.6 | 9.4 |
| 30 | 104.4753 | 77.50107 | 57.33157 |  | 30 | 14.5 | 2.9 | 13.2 |

**Raw Data**

**The viability assay of OPG**
