# Supplementary material for: In vitro evaluation of osteoprotegerin in chitosan for potential bone defect applications
Source: PeerJ. 2016 Aug 23;4:e2229. doi: 10.7717/peerj.2229 (PMC5012333; doi:10.7717/peerj.2229)
Supplement: Table S2 [file peerj-04-2229-s002.docx]

**Raw Data**

**Proliferation assay of OPG**

|  | Absorbance | | | |  | standard deviation | | | |
| --- | --- | --- | --- | --- | --- | --- | --- | --- | --- |
|  | A | B | C | D |  | A | B | C | D |
| 24 | 0.29 | 0.90 | 0.57 | 0.30 |  | 0.03 | 0.05 | 0.01 | 0.06 |
| 48 | 0.61 | 0.98 | 0.69 | 0.65 |  | 0.09 | 0.07 | 0.05 | 0.01 |
| 72 | 0.95 | 1.3 | 0.99 | 0.85 |  | 0.2 | 0.1 | 0.08 | 0.03 |
