# Supplementary material for: In vitro evaluation of osteoprotegerin in chitosan for potential bone defect applications
Source: PeerJ. 2016 Aug 23;4:e2229. doi: 10.7717/peerj.2229 (PMC5012333; doi:10.7717/peerj.2229)
Supplement: Table S3 [file peerj-04-2229-s003.docx]

**Raw Data**

**Proliferation assay of LMW chitosan combined with different concentrations of OPG**

|  | Absorbance | | | |  | standard deviation | | | |
| --- | --- | --- | --- | --- | --- | --- | --- | --- | --- |
|  | A | B | C | D |  | A | B | C | D |
| 24 | 0.71 | 1.21 | 0.88 | 0.90 |  | 0.1 | 0.26 | 0.1 | 0.06 |
| 48 | 1.27 | 1.51 | 1.057 | 1.01 |  | 0.27 | 0.08 | 0.05 | 0.2 |
| 72 | 1.3 | 1.59 | 1.127 | 1.36 |  | 0.09 | 0.11 | 0.18 | 0.08 |
