# Supplementary material for: In vitro evaluation of osteoprotegerin in chitosan for potential bone defect applications
Source: PeerJ. 2016 Aug 23;4:e2229. doi: 10.7717/peerj.2229 (PMC5012333; doi:10.7717/peerj.2229)
Supplement: Table S4 [file peerj-04-2229-s004.docx]

**Raw Data**

**Proliferation assay of MMW chitosan combined with different concentrations of OPG**

|  | Absorbance | | | |  | standard deviation | | | |
| --- | --- | --- | --- | --- | --- | --- | --- | --- | --- |
|  | A | B | C | D |  | A | B | C | D |
| 24 | 0.28965 | 0.903588 | 0.323063 | 0.482811 |  | 0.03 | 0.17 | 0.03 | 0.17 |
| 48 | 0.613597 | 0.975367 | 0.798778 | 0.635639 |  | 0.09 | 0.14 | 0.1 | 0.16 |
| 72 | 0.9478 | 1.3 | 1.0159 | 0.953111 |  | 0.2 | 0.04 | 0.22 | 0.07 |
