# Supplementary material for: In vitro evaluation of osteoprotegerin in chitosan for potential bone defect applications
Source: PeerJ. 2016 Aug 23;4:e2229. doi: 10.7717/peerj.2229 (PMC5012333; doi:10.7717/peerj.2229)
Supplement: Table S5 [file peerj-04-2229-s005.docx]

**Raw Data**

|  | Absorbance | | | |  | standard deviation | | | |
| --- | --- | --- | --- | --- | --- | --- | --- | --- | --- |
|  | A | B | C | D |  | A | B | C | D |
| 24 | 0.7056 | 0.8355 | 0.884 | 1.193 |  | 0.02 | 0.01 | 0.12 | 0.08 |
| 48 | 1.2701 | 1.36455 | 0.8965 | 1.3318 |  | 0.05 | 0.07 | 0.2 | 0.23 |
| 72 | 1.3406 | 1.3097 | 1.1601 | 1.3422 |  | 0.1 | 0.2 | 0.03 | 0.14 |

**Proliferation assay of HMW chitosan combined with different concentrations of OPG**
