# Supplementary material for: In vitro evaluation of osteoprotegerin in chitosan for potential bone defect applications
Source: PeerJ. 2016 Aug 23;4:e2229. doi: 10.7717/peerj.2229 (PMC5012333; doi:10.7717/peerj.2229)
Supplement: Table S6 [file peerj-04-2229-s006.docx]

**Raw Data**

**Proliferation assay of three different MWs of chitosan combined with 0.024 µg/mL OPG concentration using 3D culture system**

|  | Viability percentages | | |  | standard deviation | | |
| --- | --- | --- | --- | --- | --- | --- | --- |
|  | A | B | C |  | A | B | C |
| 24 | 98.5 | 89.2 | 91.3 |  | 8.3 | 20 | 25.5 |
| 48 | 110.2 | 101 | 104 |  | 9.6 | 17 | 16.2 |
| 72 | 203.9 | 169 | 173.4 |  | 17 | 22.5 | 9.1 |
