# Supplementary material for: In vitro evaluation of osteoprotegerin in chitosan for potential bone defect applications
Source: PeerJ. 2016 Aug 23;4:e2229. doi: 10.7717/peerj.2229 (PMC5012333; doi:10.7717/peerj.2229)
Supplement: Table S7 [file peerj-04-2229-s007.docx]

**Raw Data**

**Osteopontin and osteocalcin protein levels**

|  | Osteopontin | Osteocalcin |  | Osteopontin | Osteocalcin |
| --- | --- | --- | --- | --- | --- |
| 24h | 19.2575 | 23.693 |  | 0.6 | 0.6 |
| 48h | 24.714 | 25.8175 |  | 1.4 | 1.4 |
| 72h | 32.365 | 34.224 |  | 2.1 | 2.1 |
| control | 21.663 | 16.6655 |  | 2.4 | 2.4 |
